# Network analysis reveals that total hip arthroplasty restores shared segmental motion before segmental differentiation

**DOI:** 10.3389/fnetp.2026.1916345

**Published:** 2026-10-07

**Authors:** Madhur Mangalam, Brian Schlattmann, Christopher Deans, Elizabeth Wellsandt

**Affiliations:** 1 Department of Biomechanics, University of Nebraska at Omaha, Omaha, NE, United States; 2 Department of Orthopaedic Surgery & Rehabilitation, University of Nebraska Medical Center, Omaha, NE, United States; 3 Department of Health and Rehabilitation Sciences, Physical Therapy Program, University of Nebraska Medical Center, Omaha, NE, United States

**Keywords:** gait, hip osteoarthritis, network physiology, segmental differentiation, signed networks, total hip arthroplasty, whole-body coordination

## Abstract

Unilateral hip osteoarthritis (HOA) reshapes whole-body movement, and total hip arthroplasty (THA) restores function only partially—but conventional kinematic analyses fall short of characterizing how the body’s distributed coordination reorganizes. Network analyses of inter-segment kinematic coupling offer a systems-level alternative, but they conventionally collapse the sign of coupling, treating two mechanically distinct relationships as equivalent: segments whose displacement amplitudes covary, rising and falling together, and segments whose amplitudes trade off, one displacing little while the other displaces a great deal. Here, we introduce a signed partial-correlation decomposition of marker-displacement coordination into parallel covarying $\left(W^{+}\right)$ and trading-off $\left(W^{-}\right)$ networks, applied to gait from 80 healthy adults and 106 HOA patients assessed before and 6 months after THA (ClinicalTrials.gov Identifier: NCT01907503). HOA reorganizes the two networks in opposite directions: covarying coordination loses modular structure and small-worldness, while trading-off coordination becomes more locally clustered and more small-world; global efficiency falls in both. This is consistent with pain-driven splinting that consolidates a few fixed trading-off configurations. Surgery acts predominantly on covarying coordination, partially restoring its topology, while the trading-off network’s reorganization persists across the six-month post-surgical window—and on one measure, i.e., how broadly each segment spreads its trading-off partners, a deficit emerges during recovery that was absent before surgery. A node-level sign-balance metric shows that the proportion of each segment’s coordination carried by covarying relationships returns to healthy values by 6 months, even though the underlying networks remain structurally altered. Standard post-surgical care thus restores the gross balance of coordination within 6 months, while the underlying architecture remains reorganized at that mid-recovery time-point—and, on at least one measure, consolidates further. Whether this reorganization reflects protective motor strategies, and whether training targeted at segmental differentiation would reverse it, are hypotheses that the present observational data raise but cannot test.

## Introduction

Human movement requires precise and dynamic coordination across joints, muscle groups, and neural pathways (Bernstein, 1967; Latash et al., 2007; Turvey, 2007). Even simple tasks rely on the interplay of the biomechanical structure, neuromuscular control, and sensory feedback (Kelty-Stephen et al., 2021; Mangalam et al., 2023; Peterka, 2002). Yet, conventional biomechanical assessment, focused on isolated joint actions, captures little of how an injury or surgery at one joint reorganizes movement across the whole body.

This problem is the province of network physiology, which treats physiological function and state as emergent properties of dynamic interactions among systems rather than as attributes of components measured in isolation (Bashan et al., 2012; Bartsch et al., 2015; Ivanov et al., 2016; Ivanov, 2021). Its central empirical claim is that network topology carries functional meaning: the strength and organization of couplings among subsystems change systematically with the physiological state, so that transitions—across sleep stages, exercise intensities, or disease conditions—are legible as network reorganization, even when individual signals appear comparable (Bashan et al., 2012; Bartsch et al., 2015). This reframes what network analysis of movement is for. The object of interest is not a convenient summary of biomechanical variables but the coupling architecture itself, whose topology indexes integrated function. Applied to locomotion, it implies that a musculoskeletal pathology should be detectable as a change in how the body segments couple—and, importantly, that surgical resolution of the lesion need not restore the coupling architecture the pathology reorganized. Whether the topology recovers, persists, or further consolidates is then an empirical question with direct clinical relevance, and it is the question we pursue here.

Unilateral hip osteoarthritis (HOA) exemplifies this local–global divide. It is among the most prevalent degenerative joint diseases, producing chronic pain, stiffness, reduced range of motion, muscle weakness, and altered muscle activation (Grimaldi et al., 2009; Holla et al., 2011; Rutherford et al., 2015; Samaan et al., 2019; Steultjens et al., 2000), and it drives predictable cascading compensations along the kinematic chain (Eitzen et al., 2012; Foucher et al., 2007; Porta et al., 2021). These adaptations are not confined to the diseased hip; unilateral HOA is associated with bilateral deficits in dynamic balance, strength, and muscle volume (Freke et al., 2018; Loureiro et al., 2018), and they often persist after surgery (Agostini et al., 2014; Beaulieu et al., 2010; Ewen et al., 2012; Mont et al., 2007). Total hip arthroplasty (THA) reliably relieves pain and restores hip function, but the return to fluid, efficient movement is rarely immediate. Pain and patient-reported function improve substantially within the first postoperative year, whereas objective mobility can remain limited and continue to change over longer follow-up (Heiberg and Figved, 2016; Zeni et al., 2014). The conventional six-month assessment is, therefore, a mid-recovery snapshot rather than a definitive endpoint.

To capture coordination at the whole-body level, we recently introduced a network analysis of gait in HOA before and after THA (Mangalam and Kelty-Stephen, 2026), extending earlier work on healthy adults (Mangalam et al., 2026). While prior movement-network studies examined EMG-derived muscle synergies (Boonstra et al., 2015; Boonstra et al., 2016; Kerkman et al., 2018; Kerkman et al., 2020), joint kinematics (Lopez et al., 2022; Roeder et al., 2024; Romano et al., 2024), or anatomical structure (Esteve-Altava et al., 2015; Murphy et al., 2018), we built marker-based, model-free networks from the 3D displacements of reflective markers across the body. Partial correlations between marker pairs—controlling for center-of-mass (CoM) displacement to isolate inter-segment coordination from bulk translation—defined weighted graphs of segment-to-segment coupling, from which we derived local, global, and integrative graph metrics. The analysis showed fragmented coordination before surgery, partial but incomplete recovery at 6 months after THA, and lingering asymmetry, particularly in the contralateral limb (Mangalam and Kelty-Stephen, 2026). This extends the network physiology program to the level of kinematic output, complementing approaches built on physiological signals by asking whether the movement such signals generate is itself organized as an interacting network.

This framework, like most network analyses of movement, used the absolute value of inter-segment coupling. Following conventions in network neuroscience (Newman, 2010; Rubinov and Sporns, 2010), we treated edge weights as measures of coordination, collapsing positive and negative partial correlations into a single non-negative quantity. This sidesteps known difficulties with negative weights, but it obscures a mechanically critical distinction. Once CoM is regressed out, positive and negative partial correlations describe different relationships: segments whose displacement amplitudes rise and fall together (covarying coordination) versus segments whose amplitudes trade off, one displacing little while the other displaces a great deal (trading-off coordination or segmental differentiation—by which we mean differentiation of displacement amplitude between segments, not any particular functional role). Healthy gait depends on both; a magnitude-only analysis sees only their sum.

This collapse matters most where the two modes reorganize in different ways. In HOA, patients often walk with the trunk and pelvis held in stiffer, more guarded configurations (Hodges and Tucker, 2011; van Dieën et al., 2019)—plausibly a pain-avoidance strategy that minimizes displacements that provoke nociception. Such guarding could enhance covarying coordination (forcing segments into rigid covarying motion), consolidate trading-off coordination (locking the body into a few fixed trading-off relationships), or do both at once, with opposite effects on different network metrics. A magnitude-based analysis captures only the net change in coupling strength; a signed decomposition can identify which mode is responsible and whether the two recover from surgery along the same trajectory.

Here, we extend the framework with a signed partial-correlation decomposition that splits the original network into two parallel weighted graphs: a covarying network $\left(W^{+}\right)$ , retaining positive partial correlations, and a trading-off network $\left(W^{-}\right)$ , retaining the magnitudes of negative ones. Both are non-negative and feed directly into the same metrics. We also introduce a node-level sign-balance metric, $b_{i}=s_{i}^{+}/\left(s_{i}^{+}+s_{i}^{-}\right)$ , summarizing how each segment partitions its coordination between the two modes. Applying this to our previous cohort—80 healthy adults and 106 individuals with HOA assessed before and 6 months after THA (ClinicalTrials.gov NCT01907503)—we ask three questions the magnitude-based analysis could not: (i) are covarying and trading-off coordination disrupted similarly or in opposite directions? (ii) Do the two modes recover on the same trajectory, or does one lead and the other lag? (iii) Does the overall proportion of covarying to trading-off coordination return to healthy values even when the underlying network structure does not? The answers refine and, in places, overturn our earlier conclusions: HOA reorganizes the two modes in opposite directions; surgery acts predominantly on covarying coordination, while the trading-off network lags or worsens; and sign balance recovers fully even as structural deficits in both networks persist. Clinically, this indicates a more specific rehabilitation hypothesis than our earlier work could support: that standard care preferentially restores the mode that is already recovering, leaving the consolidated trading-off architecture unaddressed—a proposition that an intervention trial, not the present observational design, would settle.

## Methods

This study extends our prior network-analytic framework (Mangalam and Kelty-Stephen, 2026) by introducing a signed decomposition of the marker-displacement adjacency matrices. Participant recruitment, ethics, motion-capture acquisition, marker set, data processing, and gait-cycle segmentation are identical to those in the original analysis (Mangalam and Kelty-Stephen, 2026) and the open-access source dataset (Bertaux et al., 2022); we summarize them briefly here and detail only the methodological additions in full.

### Participants and procedure

We reanalyzed the publicly available dataset from Bertaux et al. (2022), which originated from a pre-registered clinical trial (ClinicalTrials.gov Identifier: NCT01907503; registered 25 July 2013) and was approved by the local ethics committee (CPP Est I, Dijon, France). The dataset comprises 80 asymptomatic adults (HEA; 45 women and 35 men; 58.7±15.5 y, range 25–82; 1.66±0.08 m; 69.3±13.4 kg) and 106 patients with advanced HOA (55 women and 51 men; 66.9±9.4 y, range 45–85; 1.64±0.08 m; 77.8±17.1 kg) diagnosed by the American College of Rheumatology criteria with radiological confirmation (Laroche, 2021). Patients were excluded for OA flare; painful knee, ankle, or foot disorders; acute or chronic back pain; Parkinson’s disease; neuromuscular disorders; uncontrolled diabetes; cardiac or respiratory failure; or any other condition precluding clinical gait analysis (Bertaux et al., 2022). Participants were assessed within 30 days before THA (HOA-M0) and again at 6–7 months post-surgery (HOA-M6, N=92 following attrition). All participants provided written informed consent. Overground walking trials were recorded barefoot at 100 Hz using eight optoelectronic cameras (Vicon MXT40^TM^, Vicon Motion Systems, Oxford, United Kingdom), with reflective markers placed according to the plug-in gait model (Figure 1) (Davis et al., 1991). Marker trajectories were labeled in Vicon Nexus^TM^ 2.10, interpolated via Woltring spline (Woltring, 1986), and low-pass-filtered at 10 Hz (fourth-order Butterworth). Gait events were identified using the velocity-based algorithm of Zeni et al. (2008), and gait cycles were normalized to 100% . The CoM position was computed for each cycle. The number of valid trials per participant (mean ± s.d.) was 17.61±4.06 (HEA), 15.42±3.76 (HOA-M0), and 14.45±6.95 (HOA-M6).

**FIGURE 1:**
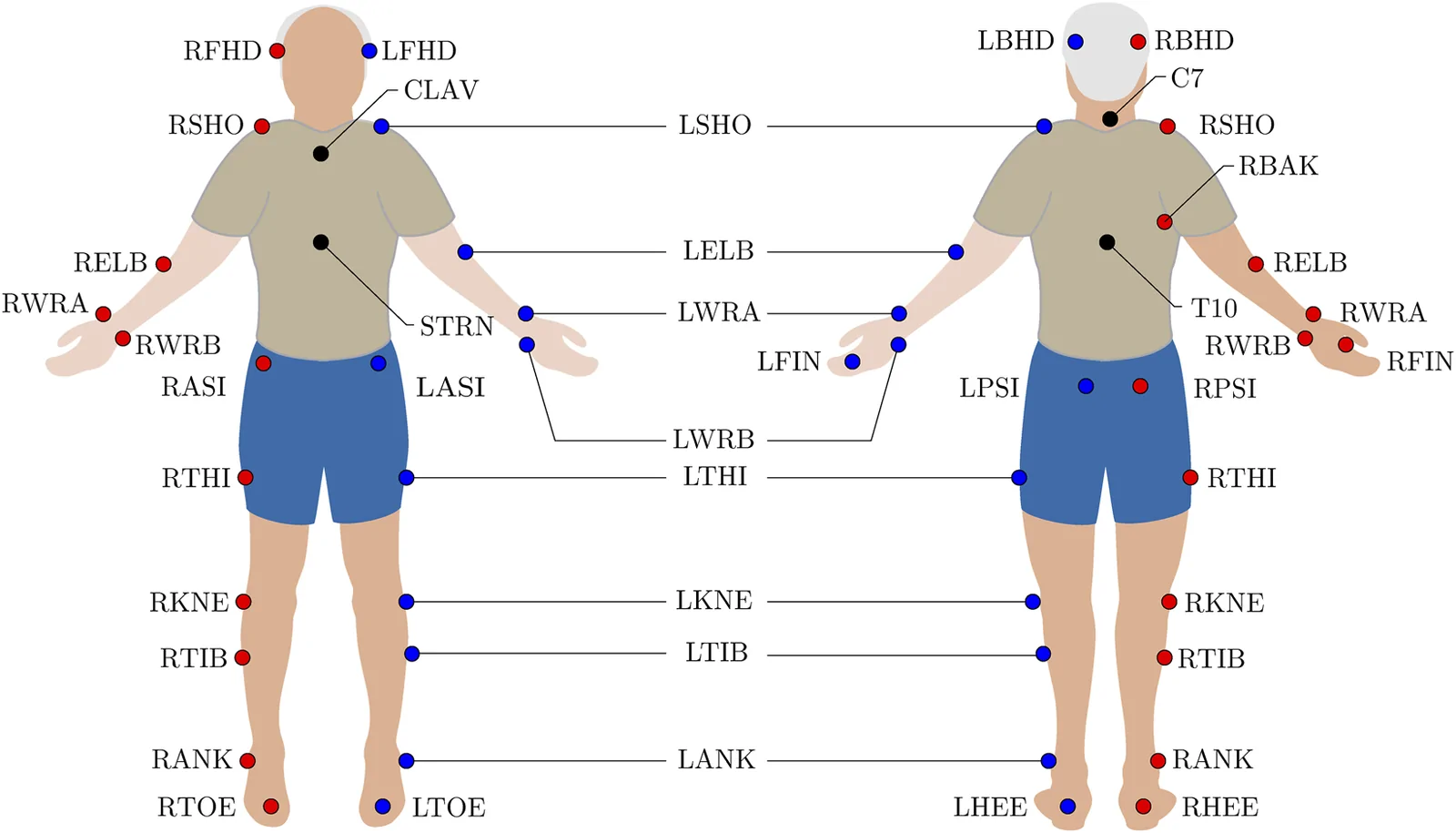
Schematic of the Vicon plug-in gait marker configuration used for motion-capture analysis. Red markers denote placements on the right side of the body, blue markers indicate left-side placements, and black markers identify midline anatomical landmarks. Marker labels follow standard plug-in gait conventions.

### Constructing signed adjacency matrices

For each trial, we computed partial correlations between the 3D Euclidean displacement time-series of every marker pair, controlling for CoM displacement. This isolates inter-segment coordination from bulk body translation. Unlike our prior analyses (Mangalam and Kelty-Stephen, 2026; Mangalam et al., 2026), which took the absolute value of these partial correlations, we retained the sign and decomposed the resulting 34×34 signed matrix $W_{\text{signed}}$ into two non-negative weighted matrices.

$$W^{+}=W_{\text{signed}}⊙I\left(W_{\text{signed}}>0\right),$$

$$W^{-}=-W_{\text{signed}}⊙I\left(W_{\text{signed}}<0\right),$$

where ⊙ denotes element-wise multiplication and I(⋅) is the indicator function. The covarying network $W^{+}$ retains pairs whose displacements covary positively after CoM motion is regressed out: when one segment moves more relative to whole-body translation, so does the other. The trading-off network $W^{-}$ retains the magnitudes of pairs whose displacements covary negatively: when one segment’s displacement is large, the other’s is small, with the roles potentially reversing across the gait cycle. This is a statement about the statistical relationship between two amplitude time-series, and several mechanical situations are compatible with it. One segment may be actively stabilized while the other moves, but low displacement can also arise passively, from proximity to an axis of rotation, from mechanical constraint, or from a segment passing through a reversal point in its trajectory while another is at peak excursion. Sequencing of this last kind is intrinsic to normal gait and implies no stabilizing role. We, therefore, use “trading-off” as a description of the coupling itself, and treat trading-off accounts as hypotheses about what generates it rather than as inferences licensed by it. Because the underlying time-series are displacement magnitudes (Euclidean distances moved), the sign of a partial correlation captures a trade-off in the amount of motion, not in its spatial direction. Two segments that displace by large amounts in opposite directions at the same instant—such as a foot and the contralateral hand swinging forward together—are both moving a great deal simultaneously and, therefore, covary positively in magnitude, placing them in $W^{+}$ rather than $W^{-}$ . The trading-off network thus encodes amplitude trade-offs between segments and does not, by construction, register directional anti-phase relationships.

Two preprocessing steps preceded network analysis. First, because each marker is trivially perfectly correlated with itself, we removed self-connections by setting the diagonal of the matrix to zero. Second, to focus on the most robust couplings and suppress weak, noise-dominated edges, we independently thresholded networks, retaining only the strongest 50% of their edges by connection magnitude. The two networks were thresholded separately so that the sparsity of one did not constrain the other.

These networks describe statistical dependencies among kinematic outputs. Because they are derived from marker displacements, they characterize the organization of movement itself rather than the neural or muscular activity that produces it: an edge indicates that the displacements of two segments covary or trade off after bulk translation is removed, not that they share neural drive or intermuscular coupling. Multiple control strategies may converge on a similar topology, and the physiological interpretations offered below are proposed as accounts consistent with the observed kinematic organization rather than as measurements of it.

### Network metrics on the covarying and trading-off networks

On each of $W^{+}$ and $W^{-}$ , we computed nine network metrics (three local, three global, and three integrative) following the definitions in our prior work (Mangalam and Kelty-Stephen, 2026). Table 1 summarizes each metric, its mathematical form, and its mechanical interpretation when applied separately to the covarying and trading-off networks. Each metric was computed on the original network and on a degree-preserving randomized counterpart generated by double-edge swaps, in which randomly selected edge pairs were rewired if doing so created neither a self-loop nor a duplicate edge, with 10 rewiring attempts per edge (Váša and Mišić, 2022); the signed difference between the two served as the dependent variable in all statistical analyses, isolating topological structure beyond what is expected from connectivity density alone.

**TABLE 1:** Network metrics computed on the covarying $\left(W^{+}\right)$ and trading-off $\left(W^{-}\right)$ networks. $s_{i}=\sum_{j}W_{ij}$ is the weighted degree (strength) of node i ; N(i) is its neighbor set; $k_{i}=|N\left(i\right)|$ ; $D_{ij}$ is the shortest-path distance computed from edge lengths $1/W_{ij}$ via Floyd–Warshall; N is the number of nodes. Full derivations are given in (Mangalam and Kelty-Stephen, 2026).

Metric	Equation	Interpretation on $W^{+}$	Interpretation on $W^{-}$
Local			
Local transitivity $T_{i}$	$T_{i}=\frac{\sum_{j,k\in N\left(i\right)}W_{jk}}{k_{i}\left(k_{i}-1\right)}$	Density of covarying coupling among segments that all covary with node i	Density of trading-off relationships among the segments that each trade off with node i ; reflects a local cluster of segments whose displacement amplitudes trade off against one another
Local efficiency $E_{i}$	$E_{i}=\frac{\sum_{j,k\in N\left(i\right)}1/D_{jk}}{k_{i}\left(k_{i}-1\right)}$	Indirect propagation of covarying motion through the local neighborhood	Indirect propagation of differentiation: How readily amplitude trade-offs chain through nearby segments
Degree centrality $D_{i}$	$D_{i}=\frac{s_{i}}{\left(N-1\right)\;W_{\max}}$	Total participation of segment i in covarying motion across the body	Total participation of segment i in trading-off relationships—the extent to which its displacement amplitude trades off against those of other segments
Global			
Modularity Q	$Q=\sum_{c}\left[\frac{L_{c}}{2m}-{\left(\frac{s_{c}}{2m}\right)}^{2}\right]$	Partition of the body into clusters of covarying segments; high $Q^{+}$ indicates fragmented covarying groups	Partition into clusters of trading-off relationships; high $Q^{-}$ indicates that differentiation is organized around a few opposing groupings
Characteristic path length L	$L=\frac{1}{\left|F\right|}\sum_{\left(i,j\right)\in F}D_{ij}$	Average reach of covarying motion across the body	Average reach of differentiation: How far a trading-off relationship propagates
Global efficiency $E_{\text{global}}$	$E_{\text{global}}=\frac{1}{N\left(N-1\right)}\sum_{i\neq j}\frac{1}{D_{ij}}$	Whole-body efficiency of covarying coordination	Whole-body efficiency of segmental differentiation
Integrative			
Global transitivity $T_{\text{global}}$	$T_{\text{global}}=\frac{\text{closed triplets}}{\text{all triplets}}$	Density of triangles of mutually covarying segments—whole-body cohesion in displacement	Density of triangles of trading-off relationships; mechanically rare in healthy gait, but informative when present
Small-worldness S	$S=\frac{T_{\text{global}}/T_{\text{random}}}{L/L_{\text{random}}}$	Balance between locally clustered and globally efficient covarying coordination	Same balance applied to differentiation: whether trading-off relationships organize both locally and globally
Participation coefficient $P_{i}$	$P_{i}=1-\sum_{s=1}^{N_{c}}{\left(\frac{\kappa_{is}}{s_{i}}\right)}^{2}$	How broadly segment i covaries across different functional modules of the body	How broadly segment i participates in trading-off relationships across multiple modules

### Community detection

Modularity Q and the participation coefficient $P_{i}$ require a partition of the network into communities. Communities were obtained by greedy modularity maximization (Newman and Girvan, 2004): each node began in its own community and was then reassigned to the neighboring community yielding the largest gain in Q , a local-moving heuristic corresponding to the first phase of the Louvain method (Blondel et al., 2008), with nodes evaluated in fixed index order and passes repeated until the change in Q fell below $10^{-6}$ or 100 passes elapsed. The procedure uses the standard Newman–Girvan modularity (resolution parameter γ=1 ) and is deterministic, so no repetition or random seeding was required; the same partition supplies the community assignments used for the participation coefficient (Guimera and Nunes Amaral, 2005). The identical procedure was applied to $W^{+}$ , $W^{-}$ , and their randomized counterparts, and partitions were estimated independently for each trial rather than imposed from a group-level template.

### Sign-balance metric

We introduce a node-level metric that quantifies each segment’s partitioning between covarying and trading-off coordination. For node i , let $s_{i}^{+}=\sum_{j}W_{ij}^{+}$ and $s_{i}^{-}=\sum_{j}W_{ij}^{-}$ denote its total covarying and trading-off strength, respectively. The sign balance is

$$b_{i}=\frac{s_{i}^{+}}{s_{i}^{+}+s_{i}^{-}}.$$

Values near 1 indicate a segment that participates almost exclusively in covarying coupling with its connected segments. Values near 0.5 indicate a balanced mix of covarying and trading-off relationships, reflecting a segment whose displacement amplitude covaries with some regions and trades off against others—the signature of segmental differentiation. Values near 0, corresponding to predominantly trading-off coordination, are mechanically rare in gait and were not observed in our data. The whole-body mean sign balance $\bar{b}=N^{-1}\sum_{i}b_{i}$ summarizes the participant’s overall partition.

### Network visualization

Networks are displayed for individual participants rather than as group averages. Averaging adjacency matrices presumes ergodicity—that a group mean network is representative of the individual networks composing it—an assumption that generally does not hold for behavioral and physiological processes (Fisher et al., 2018; Mangalam and Kelty-Stephen, 2021; Mangalam and Kelty-Stephen, 2022; Molenaar, 2004; Molenaar and Campbell, 2009). Two features of the present data make it particularly unsafe here. The cohort combines right- and left-hip pathology, and several asymmetry and internal-density effects are lateralized, with opposite signs in the two subgroups; averaging across laterality attenuates or cancels them. Furthermore, the metrics we report are non-linear functionals of the edge weights, so the modularity, clustering, and small-worldness of an averaged matrix are not the group means of those quantities, and thresholding applied after averaging retains edges consistent across participants at the expense of edges that are strong within individuals (Roberts et al., 2017). A group-average display would, thus, depict a network that is not the object of our statistical models, which are fit to trial-level networks with a random intercept by participants.

### Asymmetry and internal-density metrics

We also recomputed the asymmetry and internal-density metrics defined in our prior work (Mangalam and Kelty-Stephen, 2026) separately on $W^{+}$ and $W^{-}$ . Table 2 summarizes the definitions and their interpretations under the signed decomposition. $R_{\text{internal}}$ , $L_{\text{internal}}$ , and related submatrices are extracted from each of $W^{+}$ and $W^{-}$ and processed in parallel; $\|\cdot {\|}_{F}$ denotes the Frobenius norm, and flip(⋅) mirrors a submatrix to align anatomical orientations.

**TABLE 2:** Asymmetry and internal-density metrics under the signed decomposition. Each is computed twice, once on $W^{+}$ and once on $W^{-}$ . ϵ is a small constant to avoid division by zero.

Metric	Equation	Interpretation on $W^{+}$	Interpretation on $W^{-}$
Internal asymmetry $A_{\text{internal}}$	$\frac{\|R_{\text{internal}}-\text{flip}\left(L_{\text{internal}}\right){\|}_{F}}{\|R_{\text{internal}}{\|}_{F}+\|L_{\text{internal}}{\|}_{F}+\epsilon }$	Side-to-side imbalance in covarying coupling within each limb	Side-to-side imbalance in segmental differentiation within each limb
Cross asymmetry $A_{\text{cross}}$	$\frac{\|R_{to\text{\_}left}-\text{flip}\left(L_{to\text{\_}right}\right){\|}_{F}}{\|R_{to\text{\_}left}{\|}_{F}+\|L_{to\text{\_}right}{\|}_{F}+\epsilon }$	Imbalance in covarying coupling between sides	Imbalance in trading-off coupling between sides
Middle asymmetry $A_{\text{middle}}$	$\frac{\|R_{to\text{\_}mid}-\text{flip}\left(L_{to\text{\_}mid}\right){\|}_{F}}{\|R_{to\text{\_}mid}{\|}_{F}+\|L_{to\text{\_}mid}{\|}_{F}+\epsilon }$	Asymmetric covarying coupling with trunk/midline	Asymmetric differentiation with trunk/midline
Overall asymmetry $A_{\text{overall}}$	$\frac{A_{\text{internal}}+2A_{\text{cross}}+A_{\text{middle}}}{4}$	Composite covarying asymmetry	Composite trading-off asymmetry
Right internal density $D_{\text{right}}$	$\frac{\sum \sum \left(R_{\text{internal}}>0\right)}{n_{\text{right}}\left(n_{\text{right}}-1\right)}$	Density of covarying edges within the right limb	Density of trading-off edges within the right limb
Left internal density $D_{\text{left}}$	$\frac{\sum \sum \left(L_{\text{internal}}>0\right)}{n_{\text{left}}\left(n_{\text{left}}-1\right)}$	Density of covarying edges within the left limb	Density of trading-off edges within the left limb

### Statistical analyses

Statistical procedures are the same as in our prior analysis (Mangalam and Kelty-Stephen, 2026). For each metric, we fit a linear mixed-effects (LME) model with fixed effects of group (HEA, HOA-M0, and HOA-M6) and trial and a random intercept by a participant. To test whether the two coordination modes were affected differently by group, we additionally fit models with the coordination mode (covarying vs. trading-off) as a within-participant factor and tested the group × mode interaction. Residuals exceeding three standard deviations from the mean were removed, and models were refit. Effect sizes are reported as standardized fixed-effect coefficients $\left(\beta_{\text{std}}\right)$ with 95% confidence intervals, obtained using standardize_parameters() from the “effectsize” package (Ben-Shachar et al., 2020). Pairwise group comparisons used estimated marginal means from the “emmeans” package (Lenth et al., 2021). All analyses were performed in R (R Core Team, 2024) using lme4 (Bates et al., 2009) and lmerTest (Kuznetsova et al., 2017). The alpha level was 0.05. As in our prior analysis, we did not apply corrections for multiple comparisons: each metric tested a pre-specified hypothesis directionally grounded in the $W^{+}$ and $W^{-}$ network properties.

## Results

### Visualizing the covarying and trading-off networks

Before turning to quantitative analyses, we illustrate the structural distinction that the signed decomposition exposes. For each participant and trial, partial correlations between marker displacements (controlling for center-of-mass displacement) were separated by sign into a covarying network $W^{+}$ , retaining positive partial correlations and a trading-off network $W^{-}$ , retaining the magnitudes of negative partial correlations. Both networks are non-negative, defined on the same set of 34 anatomical markers and thresholded identically within each network. The following visualizations are illustrative rather than statistical and are presented to ground the quantitative analyses that follow. They show individual exemplars rather than group averages for the reasons given in the Methods section.

### Bodywide network visualizations across groups

Projected onto the body’s anatomical layout, the two networks reveal organizationally distinct connectivity patterns that respond differently to pathology and surgery (Figure 2). In healthy participants, the covarying network $W^{+}$ appears as a broadly distributed web of edges linking the lower limbs, trunk, and upper limbs across the body, with the strongest (darker) connections concentrated among the lower-limb and pelvis markers. The trading-off network $W^{-}$ is visibly sparser in HEA: fewer edges survive thresholding, and those that remain are of lower magnitude (yellow/orange) across most of the body. The two networks together represent two organizationally distinct layers of whole-body coordination, neither reducible to the other().

**FIGURE 2:**
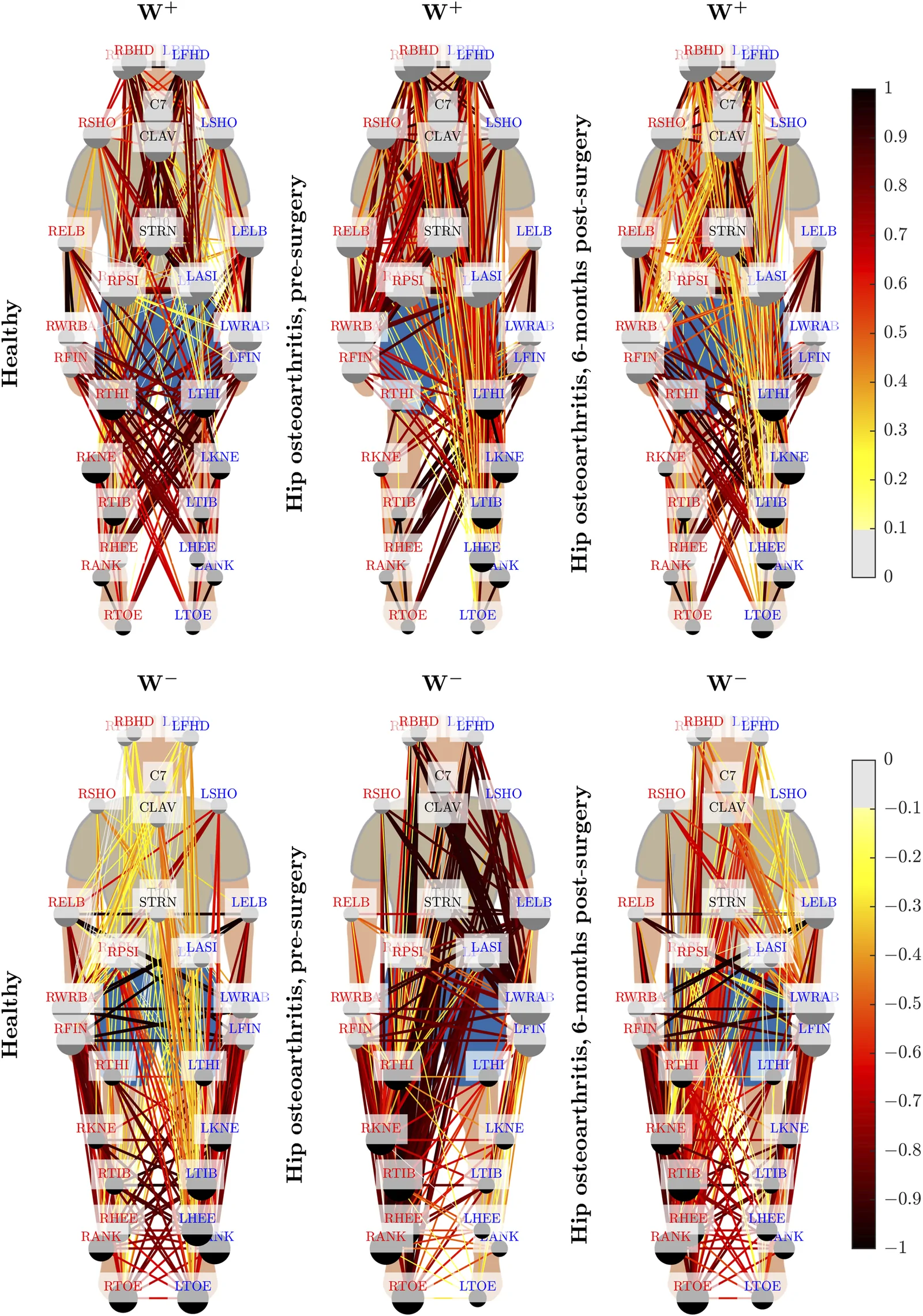
Bodywide visualizations of the covarying ( $W^{+}$ , top row) and trading-off ( $W^{-}$ , bottom row) networks for representative participants from each group. Columns show a healthy participant (HEA, left), a right-hip osteoarthritis patient pre-surgery (HOA-M0, middle), and the same patient at 6 months post-surgery (HOA-M6, right). Markers are positioned anatomically: right-side markers are shown in red, left-side markers are shown in blue, and midline markers are shown in black. Edges depict inter-segment connections retained after thresholding (top 50% by magnitude within each network), with darker shades indicating stronger connections (colorbars at right). The covarying network shows broadly distributed connections across all groups, with HOA-M6 displaying a shift toward weaker edges relative to HEA. The trading-off network is visibly sparse in HEA but densifies in HOA-M0, with high-magnitude connections concentrated in the trunk and pelvis; HOA-M6 sits between these patterns. Note: these visualizations are illustrative; group-level statistical claims are reported in the text and Table 3.

**TABLE 3:** Group × mode effects on local, global, and integrative network metrics for the covarying $\left(W^{+}\right)$ and trading-off $\left(W^{-}\right)$ networks. Each cell reports the group contrast at the corresponding coordination mode (unstandardized coefficient b , standardized coefficient $\beta_{\text{std}}$ , and p -value) derived from linear mixed-effects models with fixed effects of group, coordination mode ( $W^{-}$ reference vs. $W^{+}$ ), trial, their interaction, and a random intercept by a participant. All dependent variables are signed differences between each metric and its degree-preserving randomized counterpart. Significant increases, decreases, and null effects at p<0.05 are indicated in green, red, and gray, respectively.

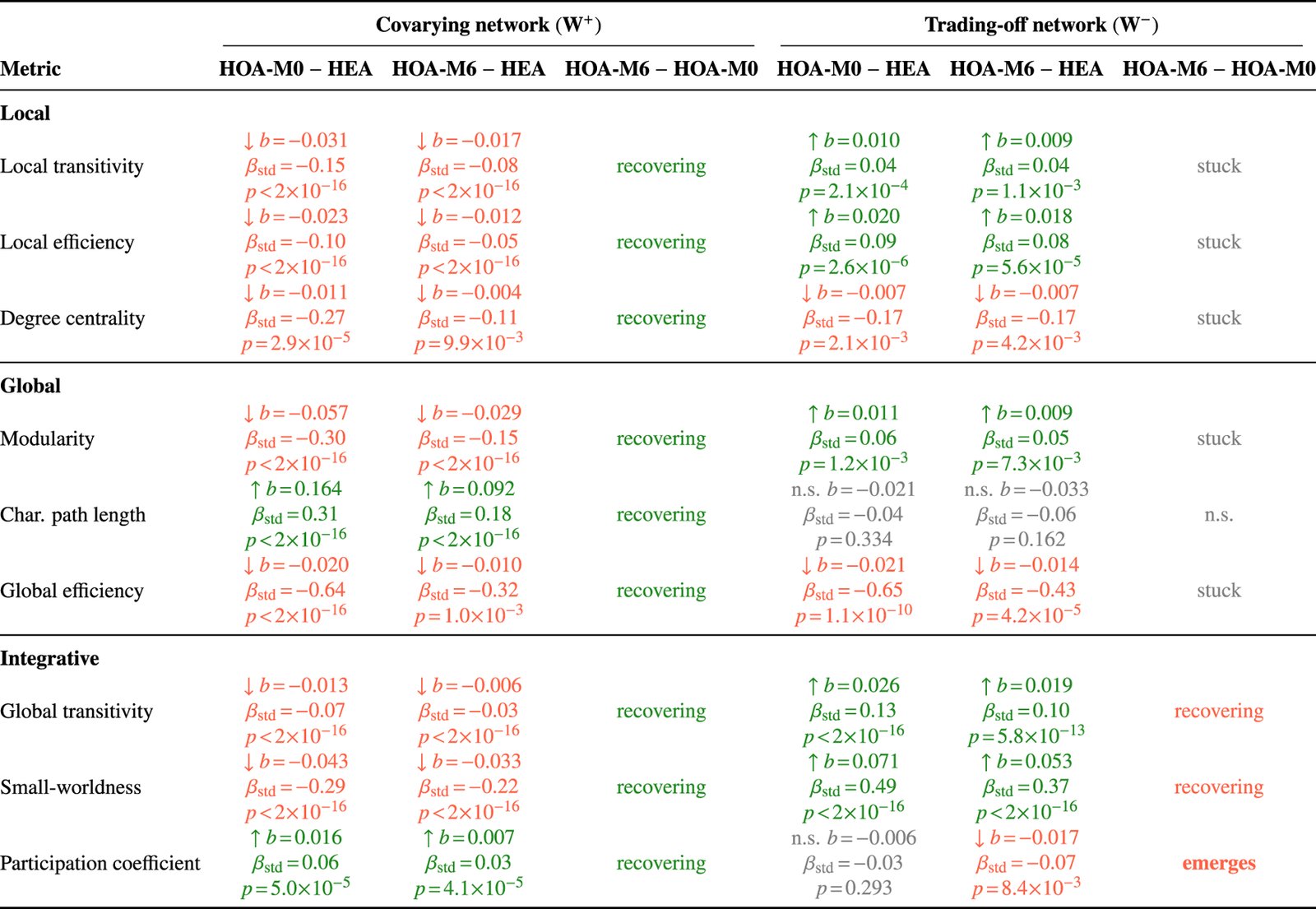

b is the unstandardized contrast; $\beta_{\text{std}}$ is the standardized fixed-effect coefficient. For $W^{-}$ , values are the group main effect (the trading-off network is the reference mode); for $W^{+}$ , values combine the group main effect with the group × mode interaction. “Recovering” indicates partial movement from HOA-M0 toward HEA; “stuck” indicates no meaningful change between HOA-M0 and HOA-M6; “emerges” indicates a deviation absent at HOA-M0 but significant at HOA-M6. Among the metrics in this table, the participation coefficient is the only one showing this pattern on the trading-off network. Colors denote the direction of the effect rather than its clinical valence: for global transitivity and small-worldness on $W^{-}$ , where HOA values are elevated relative to HEA and recovery is a decrease and is colored accordingly. All dependent variables are signed differences between each metric and its degree-preserving randomized counterpart (Váša and Mišić, 2022).

In pre-surgical HOA patients (HOA-M0), the most striking change is in the trading-off network: $W^{-}$ densifies substantially relative to HEA, with high-magnitude connections appearing across the trunk and pelvis region that were absent or weaker in healthy participants. The covarying network at HOA-M0 remains broadly distributed but exhibits subtle shifts in edge magnitude, with a more uniform spread of mid-strength connections across the body. Six months after THA (HOA-M6), the covarying network shows a shift toward weaker edges relative to HEA, indicating that the surviving covarying connections are reduced in magnitude even as their broad distribution persists. The trading-off network at HOA-M6 sits between the HEA and HOA-M0 patterns: denser than that in healthy participants, but with some reduction in the high-magnitude trunk–pelvis connections seen pre-surgery. This qualitative asymmetry—substantial trading-off densification at HOA-M0 with partial post-surgical reduction, alongside subtler covarying-network changes—previews the statistical dissociation we report below.

### Schemaball representation of network organization

To further characterize how the two networks differ in their internal organization, we constructed schemaball plots for one representative participant per group—a single healthy participant and a single right-hip osteoarthritis patient shown both before and 6 months after surgery (the same individuals depicted in Figure 2)—rather than group averages (Figure 3). Schemaballs arrange the 34 markers around a circle and draw edges as chords across the interior, exposing patterns of edge magnitude and distribution that anatomical positioning can obscure. In the healthy participant, the covarying network’s schemaball shows chords of mixed magnitude, with visible regions of lower-strength (yellow/orange) connections interleaved with stronger ones. The trading-off network’s schemaball is qualitatively similar, with substantial low-magnitude chord activity, especially among the upper-body markers.

**FIGURE 3:**
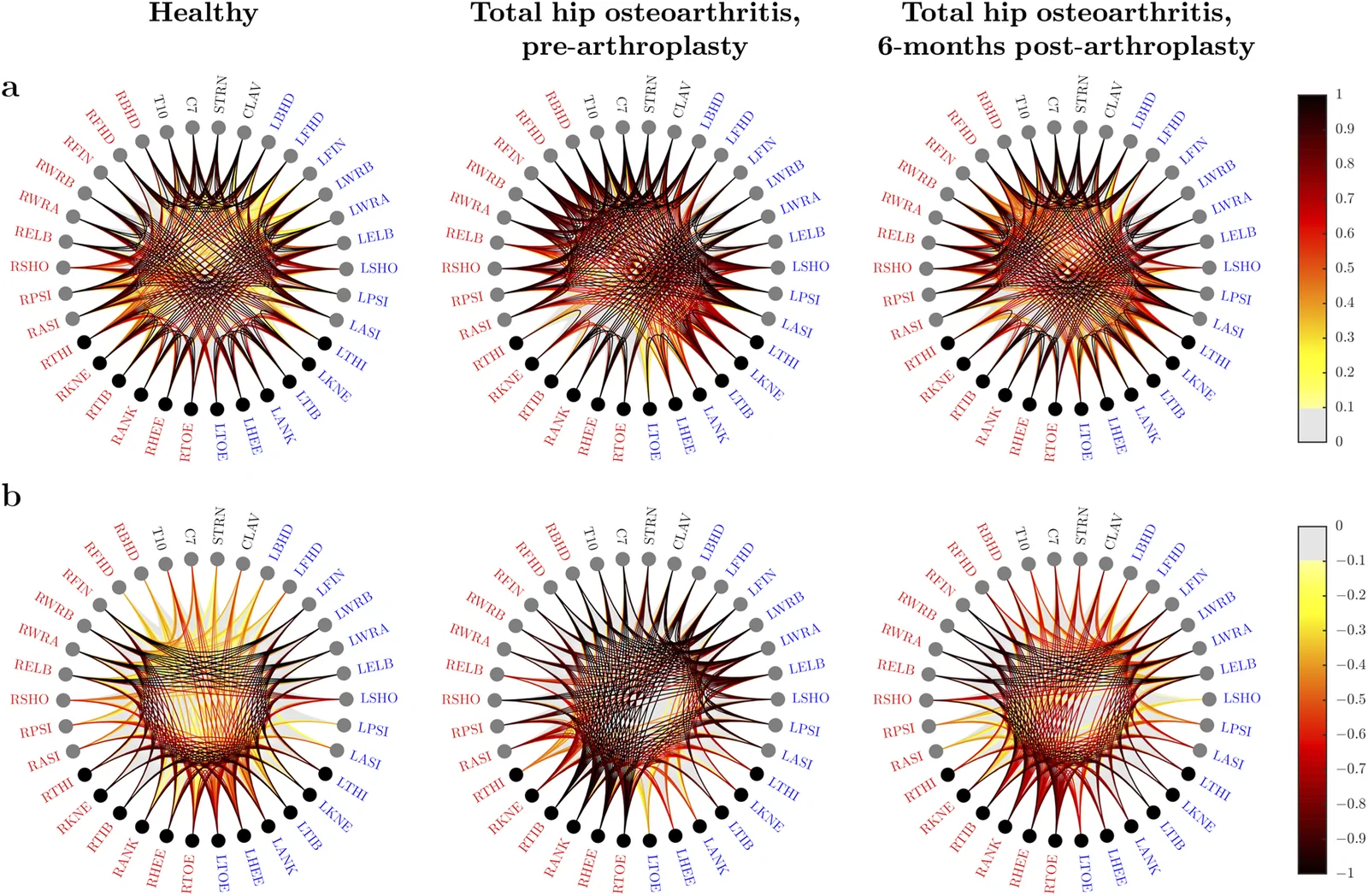
Schemaball representations of the covarying (W^+^, a) and trading-off (W^−^, b) networks for the same representative participants shown in Figure 2. The 34 anatomical markers are arranged around a circle, and inter-segment connections are drawn as chords across the interior; this layout exposes edge-magnitude and distribution patterns obscured by anatomical positioning. Columns show a healthy participant (HEA, left), a right-hip osteoarthritis patient pre-surgery (HOA-M0, middle), and the same patient 6 months post-surgery (HOA-M6, right). Right-side markers appear in red, left-side are shown in blue, and midline are shown in black; the chord shade reflects edge weight (colorbars at right), with darker shades indicating stronger connections. In the covarying network **(a)**, HEA shows chords of mixed magnitude with visible regions of lower-strength (yellow/orange) connections; both HOA panels show predominantly darker chords distributed broadly across the ring, indicating that covarying coupling becomes more uniformly strong and less differentiated in HOA, with HOA-M6 retaining much of the HOA-M0 pattern. The trading-off network **(b)** shows the clearest group differences: HEA contains many lower-magnitude chords, especially in the upper body, HOA-M0 is markedly darker and denser throughout, with most low-magnitude chords replaced by strong connections, and HOA-M6 sits between the two with some recovery of low-magnitude chords, particularly in the upper body. Note: these visualizations are illustrative; group-level statistical claims are reported in the text and Tables 3, 4.

**TABLE 4:** Group × mode effects on asymmetry and internal-density metrics for the covarying $\left(W^{+}\right)$ and trading-off $\left(W^{-}\right)$ networks. Each cell reports the group contrast at the corresponding coordination mode (unstandardized coefficient b , standardized coefficient $\beta_{\text{std}}$ , and p -value) derived from linear mixed-effects models with fixed effects of group, coordination mode, trial, and group × mode interaction, and a random intercept by a participant. Significant increases, decreases, and null effects at p<0.05 are indicated in green, red, and gray, respectively.

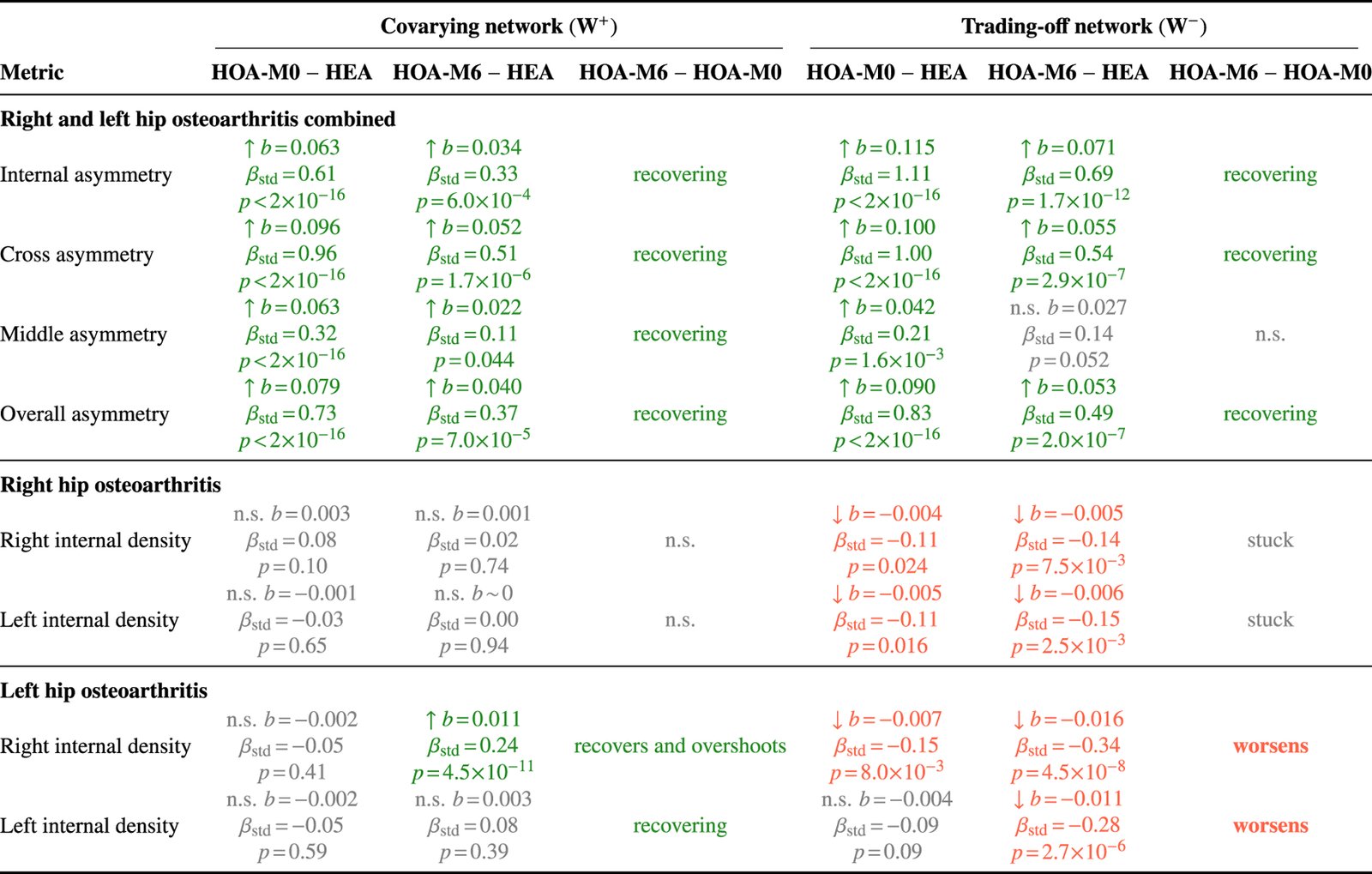

b is the unstandardized contrast; $\beta_{\text{std}}$ is the standardized fixed-effect coefficient. For $W^{-}$ , values are the group main effect (the trading-off network is the reference mode); for $W^{+}$ , values combine the group main effect with the group × mode interaction. “Recovering” indicates partial movement from HOA-M0 toward HEA; “stuck” indicates no meaningful change between HOA-M0 and HOA-M6; “worsens” indicates further deviation from HEA at HOA-M6. The contralateral-density worsening on $W^{-}$ in left-HOA patients, combined with simultaneous recovery (and overshoot) on $W^{+}$ , reveals a previously unobserved dissociation between coordination modes in unilateral compensation.

Relative to the HEA participant, both networks in HOA-M0 shift toward predominantly stronger connections throughout the whole body. The covarying schemaball loses its mix of edge magnitudes: chords become uniformly dark across the ring, indicating that covarying coupling is no longer differentiated into stronger and weaker groups. The trading-off schemaball shows the most striking change, with many low-magnitude chords of the healthy participant replaced by strong red-to-black connections distributed broadly across the body. By HOA-M6, the covarying schemaball retains much of the HOA-M0 pattern, while the trading-off schemaball shows partial movement back toward HEA, with some low-magnitude chords reappearing, especially in the upper body and trunk regions. The visual differences are subtle in any single panel but coherent across participants and consistent with the quantitative findings reported below: loss of community structure in covarying coordination, strengthening of trading-off coordination, and asymmetric recovery between the two modes.

### Marker–CoM relationships in the signed decomposition

Because both networks are constructed from partial correlations that control for CoM displacement, neither inherits the bulk-translation signal that dominates raw marker correlation. We separately examined the raw Pearson correlation between each marker’s displacement and the CoM displacement to confirm that the signed-decomposition findings reflect inter-segment coordination rather than residual bulk translation effects. Marker–CoM correlations were uniformly high across groups and showed the same group-level shift reported with the magnitude-based pipeline: HOA-M0 patients exhibited stronger marker–CoM coupling than HEA, with partial reduction at HOA-M6 (Figure 4). The CoM-controlled signed networks examined hereafter are, therefore, not confounded by this whole-body-translation effect; the coordination signatures reflect residual inter-segment dependencies after bulk body movement is removed.

**FIGURE 4:**
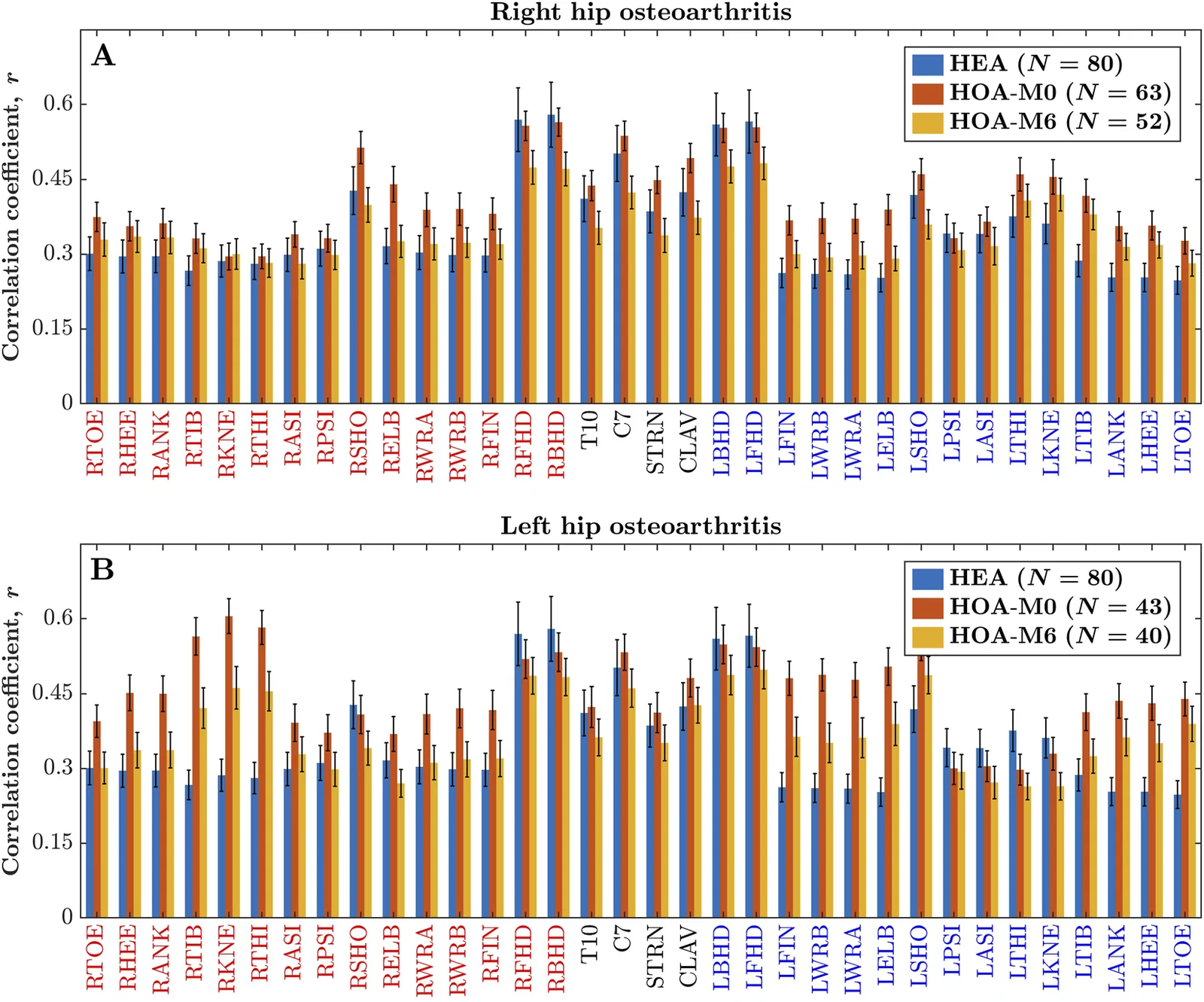
Pearson correlation coefficients (r) between marker displacement and center-of-mass (CoM) displacement for individuals with right hip osteoarthritis **(a)** and left hip osteoarthritis **(b)**. Three groups are compared: healthy adults (HEA, blue), individuals with hip osteoarthritis pre-surgery (HOA-M0, red), and the same individuals at 6 months post-surgery (HOA-M6, yellow). Markers are categorized by the anatomical location, with lower-limb markers shown in red, trunk markers shown in black, and upper-limb markers shhown in blue. Across both groups, correlations are generally lower in the post-surgical condition (HOA-M6) than in pre-surgery (HOA-M0), reflecting a shift toward the marker–CoM relationships observed in healthy individuals. This confirms that the signed covarying and trading-off networks constructed after regressing out CoM displacement are not confounded by residual bulk-translation effects. Reproduced from Mangalam and Kelty-Stephen (2026) under the CC BY-NC-ND license.

Taken together, these visualizations motivate the statistical analyses that follow: the signed decomposition does not provide two parallel versions of the same network but isolates two organizationally distinct layers of whole-body coordination, each with its own response to pathology and surgical intervention.

### Network-level assessment of whole-body coordination

HOA disrupts normal gait patterns, altering the underlying network structure of inter-segment coordination. Our prior magnitude-based analysis (Mangalam and Kelty-Stephen, 2026) demonstrated reduced global efficiency and elevated modularity in HOA, with partial recovery at 6 months after THA, but it could not distinguish whether these disruptions reflected segments moving together (covarying) or segments trading off with one another (segmental differentiation). Here, we examine each of the local, global, and integrative metrics on the covarying $\left(W^{+}\right)$ and trading-off $\left(W^{-}\right)$ networks separately (Table 3). All analyses are based on the signed difference between each network metric and its degree-preserving randomized counterpart, isolating topological structure beyond chance. HOA disrupts whole-body coordination through changes to both networks, but the two are affected differently, and the contrast is the subject of what follows.

### Local network metrics

On the covarying network, all three local metrics were significantly reduced in HOA-M0 relative to HEA, with substantial effect sizes (local transitivity $b=-0.031,\;\beta_{\text{std}}=-0.15,\;p<2\times 10^{-16}$ ; local efficiency $b=-0.023,\;\beta_{\text{std}}=-0.10,\;p<2\times 10^{-16}$ ; degree centrality $b=-0.011,\;\beta_{\text{std}}=-0.27,\;p=2.9\times 10^{-5}$ ). At HOA-M6, all three were partially attenuated but remained reduced relative to HEA $\left(-0.017\leq b\leq -0.004,\;\text{all}\;p\leq 9.9\times 10^{-3}\right)$ . The trading-off network showed a strikingly different pattern. Local transitivity and local efficiency were elevated in HOA-M0 (transitivity $b=0.010,\;\beta_{\text{std}}=0.04,\;p=2.1\times 10^{-4}$ ; efficiency $b=0.020,\beta_{\text{std}}=0.09,\;p=2.6\times 10^{-6}$ ), while degree centrality was slightly reduced $\left(b=-0.007,\;\beta_{\text{std}}=-0.17,\;p=2.1\times 10^{-3}\right)$ . Crucially, none of these trading-off-network deviations changed meaningfully between HOA-M0 and HOA-M6 (all pairwise p≥0.99 ), indicating that the local-level reorganization of trading-off coordination remains unchanged across the recovery window. No main effect of trial reached significance for any local metric for either network (all p≥0.49 ).

### Global network metrics

The global metrics revealed two distinct patterns. Modularity and characteristic path length followed the opposite-direction changes present in the local metrics. Covarying-network modularity was substantially reduced in HOA-M0 $\left(b=-0.057,\;\beta_{\text{std}}=-0.30,\;p<2\times 10^{-16}\right)$ , reflecting a loss of the normal community structure in covarying coordination: segments that should cluster into distinct covarying groups collapsed into a single broadly covarying block. Characteristic path length on the covarying network was elevated $\left(b=+0.164,\;\beta_{\text{std}}=+0.31,\;p<2\times 10^{-16}\right)$ , indicating that covarying signals had to traverse longer paths to connect distant segments. The trading-off network showed an opposite-direction pattern for modularity. It was slightly elevated at HOA-M0 $\left(b=0.011,\;\beta_{\text{std}}=0.06,\;p=1.2\times 10^{-3}\right)$ but was unaffected on the characteristic path length at either time-point (both p≥0.16 ). Both covarying-network metrics recovered partially by HOA-M6 (modularity halving to $\beta_{\text{std}}=-0.15$ ; characteristic path length reducing by approximately 45% to $\beta_{\text{std}}=+0.18$ ; both $p<2\times 10^{-16}$ ), while the trading-off-network modularity deficit remained stuck ( $b=0.009,\;p=7.3\times 10^{-3}$ at HOA-M6).

Global efficiency broke this opposite-direction pattern: it was strongly reduced in HOA-M0 for both networks, with comparably large effect sizes (covarying $b=-0.020,\;\beta_{\text{std}}=-0.64,\;p<2\times 10^{-16}$ ; trading-off $b=-0.021,\;\beta_{\text{std}}=-0.65,\;p=1.1\times 10^{-10}$ ). At HOA-M6, the covarying-network deficit recovered substantially (to $\beta_{\text{std}}=-0.32,\;p=1.0\times 10^{-3}$ ), while the trading-off-network deficit reduced numerically but remained large and statistically indistinguishable from that at HOA-M0 (pairwise contrast p=0.21 ). For global efficiency, then, the dissociation lies not in the direction of disruption but in the trajectory of recovery—a pattern also seen for degree centrality.

### Integrative network metrics

The integrative metrics revealed the strongest and most consistent dissociation between the two networks. Global transitivity decreased for the covarying network in HOA-M0 $\left(b=-0.013,\;\beta_{\text{std}}=-0.07,\;p<2\times 10^{-16}\right)$ but increased for the trading-off network $\left(b=0.026,\;\beta_{\text{std}}=0.13,\;p<2\times 10^{-16}\right)$ , which is a direct mechanical opposition. Small-worldness showed the same opposite-direction pattern with the largest standardized effect sizes in the entire analysis: covarying $b=-0.043,\;\beta_{\text{std}}=-0.29,\;p<2\times 10^{-16}$ ; trading-off $b=+0.071,\beta_{\text{std}}=+0.49,\;p<2\times 10^{-16}$ . At HOA-M6, both directions of effect persisted, but with reduced magnitudes (covarying small-worldness $\beta_{\text{std}}=-0.22$ ; trading-off $\beta_{\text{std}}=+0.37$ ; both $p<2\times 10^{-16}$ ). Taken together, these findings indicate that HOA reorganizes the body’s covarying coordination away from its normal small-world architecture (less locally clustered and less efficiently integrated) while simultaneously consolidating trading-off coordination into a more pronounced small-world configuration: tighter local clusters of segments that trade off with one another, with reduced reach across the rest of the body.

The participation coefficient was the only integrative metric to show a substantively different pattern and yielded the analysis’s most striking finding. On the covarying network, participation was elevated in HOA-M0 $\left(b=+0.016,\;\beta_{\text{std}}=+0.06,\;p=5.0\times 10^{-5}\right)$ , reflecting that covarying connections in HOA were spread too broadly across functional modules—segments participated in covarying motion with everything rather than with specific functional groups, which is consistent with the loss of modular structure noted above. By HOA-M6, the elevation reduced toward HEA values. The trading-off network showed the opposite pattern with a striking temporal twist. At HOA-M0, trading-off participation was statistically indistinguishable from HEA $\left(b=-0.006,\;\beta_{\text{std}}=-0.03,\;p=0.29\right)$ . By HOA-M6, however, the deficit emerged: trading-off participation dropped significantly below HEA $\left(b=-0.017,\;\beta_{\text{std}}=-0.07,\;p=8.4\times 10^{-3}\right)$ . Among the nine network metrics, this is the only one where the HOA deviation emerges between M0 and M6 rather than recovering or remaining stuck; a comparable pattern appears in the laterality-specific internal densities reported below. Mechanistically, this indicates that during post-surgical adaptation, trading-off relationships became more locally concentrated rather than distributed across functional modules: segments engaging in trading-off coordination did so with an increasingly narrow set of partners as recovery progressed. The same dissociation resolves an apparent paradox in our prior magnitude-based analysis (Mangalam and Kelty-Stephen, 2026), where the participation coefficient unexpectedly decreased post-surgery; the signed decomposition reveals that this decrease was driven specifically by the trading-off network and was caused by partial recovery of the covarying network.

### Summary of network-level alterations

Across all nine metrics, every covarying-network effect is large and partially recovers by 6 months, whereas the trading-off network’s disruptions take three forms. For the clustering metrics (local transitivity, local efficiency, modularity, global transitivity, and small-worldness), the trading-off network shows smaller but consistent opposite-direction effects, indicating that HOA actively reorganizes trading-off coordination into a more compact, locally clustered configuration. For global efficiency and degree centrality, the trading-off network shows same-direction reductions comparable in magnitude to the covarying-network deficits but fails to recover with surgery. For the participation coefficient, the trading-off-network deficit appears only after surgery. Across all three forms, the covarying network responds to THA but the trading-off network does not.

### Asymmetry reduction over 6 months but with lingering functional deficits, distributed differently across the coordination modes

Hip osteoarthritis often presents unilaterally and is associated with chronic biomechanical adaptations, including altered joint loading, pain-avoidance behaviors, and compensatory gait changes. Because these factors lead to pronounced side-to-side differences, individuals with unilateral OA may experience differential adaptation in both the affected and contralateral hips, leading to variations in movement asymmetry compared to that in healthy controls. Our prior magnitude-based analysis (Mangalam and Kelty-Stephen, 2026) showed elevated asymmetry pre-surgery with partial recovery at 6 months after THA, but it could not address whether the lateral imbalance was present in segments that covary in displacement, in segments that trade off, or in both. The signed decomposition resolves this question by examining each asymmetry and internal-density metric on the trading-off $\left(W^{-}\right)$ and covarying $\left(W^{+}\right)$ networks separately (Table 4).

### Asymmetry is elevated on both networks and partially recovers on both

All four asymmetry metrics (internal, cross, middle, and overall) were significantly elevated in HOA-M0 compared with HEA across both networks. Effect sizes were large to very large on the trading-off network $\left(0.21\leq \beta_{\text{std}}\leq 1.11,\;\text{all}\;p\leq 1.6\times 10^{-3}\right)$ , with internal asymmetry showing the largest disruption $\left(\beta_{\text{std}}=1.11,\;p<2\times 10^{-16}\right)$ . On the covarying network, effects ranged from moderate to very large $\left(0.32\leq \beta_{\text{std}}\leq 0.96,\;\text{all}\;p<2\times 10^{-16}\right)$ , with cross asymmetry showing the strongest disruption $\left(\beta_{\text{std}}=0.96\right)$ . By 6 months post-surgery, asymmetry remained significantly elevated relative to HEA across all four metrics on both networks (Table 4), but with markedly attenuated effect sizes (trading-off: $0.14\leq \beta_{\text{std}}\leq 0.69$ ; covarying: $0.11\leq \beta_{\text{std}}\leq 0.51$ ). The recovery was statistically robust on both networks: within the HOA cohort, asymmetry decreased significantly from M0 to M6 on internal, cross, and overall asymmetry across both $W^{-}$ and $W^{+}$ ( allp≤0.0008 ; pairwise contrasts shown in Table 4).

### Middle asymmetry is the exception—a covarying-dominant disruption

Middle asymmetry, which quantifies side-to-side imbalance in how the two limbs couple to midline structures (trunk, pelvis, and head), behaved differently from the other three metrics. The trading-off network showed only a modest pre-surgical effect $\left(\beta_{\text{std}}=0.21,\;p=1.6\times 10^{-3}\right)$ that was not significant at M6 $\left(\beta_{\text{std}}=0.14,\;p=0.052\right)$ . The covarying network showed a substantially larger pre-surgical disruption ( $\beta_{\text{std}}=0.32,\;p<2\times 10^{-16}$ ; group × mode interaction $\beta_{\text{std}}=0.11,\;p=6.6\times 10^{-5}$ ) that recovered partially at M6 $\left(\beta_{\text{std}}=0.11,\;p=0.044\right)$ . The cohabitation of these patterns localizes the asymmetry burden in HOA to covarying coordination with the trunk–pelvis–head region. One candidate explanation is pain-driven splinting biasing trunk and pelvis displacement toward the unaffected side; because no pain or muscle-activation measure was part of our analysis, this remains a hypothesis that the present data motivate rather than confirm. The trading-off network, in contrast, distributes its midline-related asymmetry symmetrically across both sides regardless of pathology.

### Laterality-specific changes in internal density reveal coordination-mode dissociation

The internal density of connections differed both by the laterality of hip OA and by the coordination mode. In right-hip OA patients, both right and left trading-off densities were reduced relative to HEA, and these reductions did not recover over 6 months (right: HOA-M0 $\beta_{\text{std}}=-0.11,\;p=0.024$ ; HOA-M6 $\beta_{\text{std}}=-0.14,\;p=7.5\times 10^{-3}$ ; left: HOA-M0 $\beta_{\text{std}}=-0.11,\;p=0.016$ ; HOA-M6 $\beta_{\text{std}}=-0.15,p=2.5\times 10^{-3}$ ). The covarying densities showed no significant group effects at either time-point (all p≥0.10 ).

In participants with left-hip OA, the pattern was more marked and revealed a previously unobserved dissociation. On the trading-off network, contralateral (right) internal density showed a modest deficit at M0 $\left(\beta_{\text{std}}=-0.15,\;p=8.0\times 10^{-3}\right)$ that more than doubled by M6 $\left(\beta_{\text{std}}=-0.34,\;p=4.5\times 10^{-8}\right)$ . The ipsilateral (left) trading-off density followed the same pattern, deteriorating from a non-significant deficit at M0 $\left(\beta_{\text{std}}=-0.09,\;p=0.09\right)$ to a robust deficit at M6 $\left(\beta_{\text{std}}=-0.28,\;p=2.7\times 10^{-6}\right)$ . Notably, the same participants demonstrated simultaneous overshoot recovery on the covarying network: contralateral right covarying density increased to above-HEA levels by M6 (group × mode interaction $\beta_{\text{std}}=0.58,\;p=4.5\times 10^{-11}$ ), and ipsilateral left covarying density recovered toward HEA values (interaction $\beta_{\text{std}}=0.36,\;p=1.8\times 10^{-5}$ ).

This dissociation indicates that the period from M0 to M6 in left-hip OA patients does not show a uniform recovery: the body is simultaneously increasing covarying connectivity within both limbs while decreasing trading-off connectivity within both limbs. Mechanistically, this represents a trade-off between the two coordination modes during post-surgical adaptation. Within each limb, segments come to displace together more cohesively while amplitude differentiation among them declines. Whether this reflects a learned adaptation or a passive consequence of altered loading cannot be determined from displacement statistics alone. The right-hip OA group did not show this dissociation pattern, indicating that compensation dynamics following unilateral hip replacement may differ by the side of pathology—a possibility that our prior magnitude-based analysis could not have detected. Limb dominance was not recorded in the source dataset, so whether this laterality effect tracks dominance cannot be determined here. The trial effects on asymmetry metrics were small $\left(\beta_{\text{std}}\leq 0.02\right)$ but reached significance for internal, cross, and overall asymmetry (p≤0.035) , reflecting modest session-to-session adaptation across the protocol.

### Summary of alterations in asymmetry and internal density

The signed decomposition refines the asymmetry pattern in two important and clinically meaningful ways. First, lateral imbalance in HOA is not confined to one coordination mode: both the trading-off and covarying networks show elevated asymmetry that partially recovers over 6 months. The trading-off network carries the larger absolute disruption on internal and overall asymmetry, while the covarying network carries the disruption on midline-related asymmetry, identifying trunk–pelvis splinting as a covarying phenomenon. Second, internal density reveals an unexpected dissociation: in left-hip OA patients, the contralateral and ipsilateral trading-off densities deteriorate further during the recovery window even as the corresponding covarying densities recover or overshoot. This pattern indicates that standard post-surgical adaptation reinforces cohesive within-limb covarying coupling at the cost of segmental differentiation, leaving patients with denser-but-less-differentiated coordination within each limb. Such a trade-off is invisible to magnitude-based network analyses. We raise it as a candidate target for differentiation-focused training, while noting that no intervention was tested here and that the therapeutic relevance of this pattern is at present a conjecture.

### Sign balance: an integrative signature that recovers fully

The trading-off and covarying networks indicate complementary but separate stories about HOA’s effect on whole-body coordination. To examine the body’s overall partition between the two coordination modes, we computed the node-level sign-balance metric $b_{i}=s_{i}^{+}/\left(s_{i}^{+}+s_{i}^{-}\right)$ —the proportion of each segment’s total coordination strength carried by covarying relationships—and averaged it across the body. Values near unity indicate a body whose coordination is dominated by covarying coupling; values near 0.5 indicate balanced contribution from both modes; values near zero indicate the dominance of trading-off coordination. Healthy adults were in the intermediate range characteristic of differentiated whole-body movement $\left({\bar{b}}_{\text{HEA}}=0.583\right)$ .

HOA shifted this balance downward in a small but statistically significant way. Pre-surgical patients showed reduced sign balance relative to HEA ( ${\bar{b}}_{\text{HOA}-\text{M0}}=0.576$ ; $b=-0.0066,\;\beta_{\text{std}}=-0.33\;\left[-0.56,-0.09\right],\;p=6.6\times 10^{-3}$ ), indicating that segments in HOA-M0 carried proportionally more trading-off coordination per node than they did in healthy controls. Mechanistically, this is consistent with the ${\text{W}}^{-}$ network results: trading-off relationships became more locally clustered, more small-world, and more transitively organized in HOA, so the proportion of each segment’s coordination carried by trading-off relationships increased even as covarying coordination collapsed in modularity and small-worldness. The shift is small in absolute terms (˜1% of the HEA mean) but moderate in standardized terms, and it is pre-specified directionally by the ${\text{W}}^{+}$ and ${\text{W}}^{-}$ analyses.

Six months after THA, sign balance returned to values statistically indistinguishable from HEA $\left(b=-0.0009,\;\beta_{\text{std}}=-0.05\;\left[-0.30,\;0.21\right],\;p=0.72\right)$ . Pairwise comparison within the HOA cohort confirmed a significant increase from M0 to M6 ( b=+0.0057,p=0.047 Tukey-adjusted). No main effect of trial was observed (p=0.50) .

Among the network metrics, sign balance is the only one for which HOA-M6 recovered fully to HEA values. Alongside the network-level and asymmetry findings—both of which show persistent residual deficits at HOA-M6—this dissociation is informative rather than contradictory. The whole-body proportion of covarying to trading-off coordination restores by 6 months; however, the structure of each network does not. These are logically independent: because $b_{i}$ is a ratio, it can return to healthy values, while both the magnitude and the topology of coupling remain altered, which is what we observe. An individual at HOA-M6 has approximately the correct ratio of covarying to trading-off coordination per segment, while both networks retain the structural deficits reported above. Thus, sign balance serves as a coarse-grained recovery indicator, whereas the separated $W^{+}$ and $W^{-}$ analyses retain their sensitivity to what a summary metric conceals.

## Discussion

Six months after hip replacement, patients walk with restored stride length, symmetric timing, and full range of motion—yet, by current orthopedic standards, 6 months is the middle, not the end, of recovery, with patient-reported outcomes, strength, and activity participation continuing to improve through 12–24 months. Our prior network analysis (Mangalam and Kelty-Stephen, 2026) showed that this surface recovery masks persistent coordination deficits invisible to conventional gait measures. The present results sharpen that picture. Decomposing inter-segment coupling into covarying and trading-off components reveals that the residual deficit is not a uniform shortfall in coordination strength but a specific reorganization—one that leaves the overall proportion of the two modes restored while the structure of each network remains altered.

This dissociation matters because the two modes carry different mechanical functions. Covarying coupling reflects segments displacing together: a shared response to loading and common entrainment to gait rhythm. Trading-off coupling reflects amplitude differentiation between segments: the trunk displacing little while the pelvis displaces more and an arm swinging while the contralateral leg is in stance. Whether a given trade-off arises from active stabilization or from the ordinary phasing of a cyclical task is not settled by the correlation structure alone; what the statistic establishes is that the two amplitudes are differentiated rather than shared. A healthy gait needs both. In HOA, covarying modularity and small-worldness collapsed ( $\beta_{\text{std}}=-0.30$ and −0.29 ), as segments that should form distinct covarying groups became broadly intermixed. The trading-off network developed the opposite signature—elevated clustering, transitivity, and small-worldness ( $\beta_{\text{std}}$ ranging from 0.04 to 0.49). This is not a loss of trading-off coordination but a consolidation of it into a tighter, more locally interconnected configuration, consistent with pain-driven splinting that restricts differentiation to a few fixed trading-off pairings.

The asymmetric recovery admits a plausible but untested interpretation, which we offer as a hypothesis for future work rather than as a mechanism that our data establish. Covarying coordination may track the resolution of the local mechanical problem: as pain abates and the joint accepts load, segments previously constrained to rigid covarying motion could regain their capacity for covarying displacement. Testing this would require pain and loading measures alongside the kinematics, which the present dataset lacks. The persistence of the trading-off pattern, by contrast, is consistent with a slower behavioral process: the relearning of relative motion between segments that pain had trained the patient to move as a unit. Our data establish that the kinematic pattern persists, not that this mechanism produces it. Pain resolution removes the original driver, but the motor habit outlives the pain that produced it (Sterling et al., 2001; Hodges and Smeets, 2015). The trading-off network may be the kinematic signature of exactly this persistence.

The participation coefficient warrants particular attention, as in our magnitude-based analysis (Mangalam and Kelty-Stephen, 2026), it decreased between pre- and post-surgery rather than recovering—an anomaly that the signed decomposition resolves. Covarying participation behaved as expected—elevated at HOA-M0 and partially restored by HOA-M6. However, on the trading-off network, the deficit emerged during recovery rather than being present from the start: segments engaging in trading-off coordination did so with an increasingly narrow set of partners as recovery progressed. Among the nine network metrics, this is the only one in which the HOA deviation emerged over the post-surgical window, indicating that standard post-THA care, while driving substantial covarying recovery, may simultaneously consolidate coordination into more compact configurations.

Sign balance offers the integrative counterpoint. Pre-surgically, it shifted downward as segments carried proportionally more trading-off coordination per node—a direct consequence of trading-off consolidation combined with covarying collapse. By HOA-M6, it returned to values statistically indistinguishable from HEA, the only metric in the analysis to recover fully. The lesson is interpretive: a ratio measure can normalize while the architecture it summarizes remains altered. A patient whose sign balance has returned to healthy values might still move with consolidated trading-off coordination and reduced covarying modularity—the gross balance is restored, but the fine structure is not.

Clinically, this sharpens the rehabilitation hypothesis, though not yet the prescription. Standard post-THA protocols emphasize strengthening, range of motion, and symmetric bilateral exercises—activities plausibly aligned with covarying coupling, the mode in which patients are already recovering. Comparatively little targets amplitude differentiation between segments. We emphasize that this correspondence between protocol content and coordination mode is inferred rather than measured: we did not record what rehabilitation these patients received and cannot attribute the observed recovery pattern to it. Exercises that explicitly target segmental differentiation—trunk–pelvis dissociation drills, split-stance work with contralateral reach, step-ups with opposite-arm drive, reciprocal arm-swing retraining during overground walking—map naturally onto the deficits we observe. Because the trading-off participation deficit emerges during the post-surgical window rather than being present beforehand, timing may matter: differentiation exercises might be introduced during recovery rather than after it. This is a hypothesis drawn from a coincidence in time—our data show that consolidation occurs while patients are under standard care, not that standard care produces it, nor that earlier intervention would prevent it. Whether continued standard care between six and 24 months eventually reverses this consolidation, or whether earlier targeted intervention accelerates its reversal, is an empirical question the present data cannot resolve.

More broadly, these findings show why network analysis is a fundamentally different level of description, not merely a different visualization of conventional gait variables. Traditional analyses decompose gait into isolated quantities—joint angles, ranges of motion, peak velocities, and stride timing—that can tell whether the hip flexes less or cadence slows, but it cannot show how coordination reorganizes across the body as an interacting system. The phenomenon here is inherently relational: it lives in the structure of statistical dependencies among dozens of simultaneously moving segments, not in any single joint. Two patients could share identical hip range of motion, stride length, and walking speed, yet differ profoundly in whether their segments remain coordinated as a differentiated system or have collapsed into a more compact configuration. The signed decomposition further sharpens this relational advantage: even multivariate methods such as principal component analysis (Deluzio and Astephen, 2007; Federolf et al., 2013) or gait-profile scores (Baker et al., 2009; Schwartz and Rozumalski, 2008) reduce movement to dominant variance patterns without preserving inter-segment topology, and magnitude-only networks—including our own prior analysis (Mangalam and Kelty-Stephen, 2026)—could detect that coordination remained abnormal after surgery but could not show why it persisted. Only the signed framework localizes the residual deficit to a specific persistence of consolidated trading-off organization, with a deficit in trading-off participation emerging during the post-surgical window itself.

These results connect to, and depart from, three strands of prior network work on movement. Muscle-network studies built from EMG coherence and synergy decompositions have established that the motor system is organized into functionally distinct subnetworks whose structure is constrained by musculoskeletal anatomy across multiple time-scales (Boonstra et al., 2015; Boonstra et al., 2016; Kerkman et al., 2018) and that synergy-based and coherence-based networks capture different modes of coordination within the same walking task (Kerkman et al., 2020). A complementary physiological-network framework quantifies inter-muscular coupling through amplitude–amplitude cross-frequency coupling (ACFC), correlating the spectral power dynamics of EMG frequency bands across muscles to expose networks of synchronous muscle fiber activation that reorganize with fatigue (Garcia-Retortillo and Ivanov, 2022; Garcia-Retortillo et al., 2023) and degrade with age (Garcia-Retortillo et al., 2025). ACFC and the signed decomposition unpack coordination along orthogonal axes—the former resolving physiological coupling by frequency band and the latter resolving kinematic coupling by the mechanical sign of the dependency—and neither substitutes for the other.

Similarly to the EMG-based frameworks above, our covarying and trading-off networks treat coordination as multi-modal, but at the kinematic level: whole-body coordination is not a single quantity but a superposition of modes that can be dissociated, and—this is the extension—that can reorganize in opposite directions under a single pathology and recover on different trajectories after a single intervention. Where the EMG literature separates modes by frequency band or decomposition method, the signed decomposition separates them by the mechanical sign of the dependency itself, requiring no additional signal, model, or basis choice. The two approaches are complementary and, in principle, jointly testable: if covarying edges reflect common drive to segments loaded together, and trading-off edges reflect reciprocal organization between segments whose displacement amplitudes alternate, then $W^{+}$ and $W^{-}$ should map onto separable intermuscular coherence structures—a prediction that concurrent EMG and motion capture could adjudicate. Anatomical network analyses (Esteve-Altava et al., 2015; Murphy et al., 2018) address the same question from the opposite direction, defining the structural backbone that determines which trade-off relationships are mechanically available in the first place. Prior kinematic network studies (Lopez et al., 2022; Roeder et al., 2024; Romano et al., 2024) are the closest methodological antecedents of the present work and, like our own earlier analysis (Mangalam and Kelty-Stephen, 2026), use magnitude-valued coupling. Our results imply a specific prediction for those datasets: wherever covarying and trading-off coupling shift in opposition, magnitude-based edge weights will partially cancel, and the reported group effects will be attenuated rather than absent. Small or null effects in magnitude-based kinematic networks should, therefore, be re-examined under a signed decomposition before being interpreted as preserved coordination.

The aging literature bears on our group contrast in a way that is worth making explicit, since our HOA cohort is, on average, older than the healthy comparison group. Aging alters neuromuscular control in a characteristic direction: older adults have reduced complexity and dimensionality of muscle coordination during walking (da Silva Costa et al., 2020) and draw on a narrower motor repertoire when responding to balance challenges (Allen and Franz, 2018), a pattern that parallels the neural dedifferentiation reported in aging more broadly (Koen and Rugg, 2019). If our findings were driven primarily by age, the expected signature would be a global loss of differentiation: uniform weakening of the trading-off network, with covarying coordination correspondingly dominant and topology largely preserved. That is not what we observe. Trading-off coordination in HOA is not diminished but restructured—more locally clustered, more transitive, and more small-world ( $\beta_{\text{std}}$ up to 0.49)—while its degree centrality falls and its global efficiency drops in parallel with the covarying network. Elevated local organization alongside reduced global reach is a reorganization signature, not an attrition signature, and it is not what generalized age-related dedifferentiation predicts. The within-subject M0-to-M6 contrast, in which age is held effectively constant, reinforces this reading: the covarying network recovers substantially over 6 months, while the trading-off network remains stuck or worsens, a dissociation that no account based on chronological age can produce. The age difference nonetheless remains a limitation of the cross-sectional comparison, which we return to below.

Several limitations apply. First, the six-month window cannot speak to longer-term adaptation trajectories, leaving open whether trading-off coordination eventually recovers, plateaus, or continues to consolidate—a caveat that bears directly on the participation-coefficient finding, arguably our most consequential single result, which is statistically robust and mechanistically interpretable but drawn from a single cohort and a single recovery window. Second, the cross-sectional contrast with healthy adults, rather than longitudinal pre-pathology data, cannot establish whether some component of the observed reorganization reflects pre-existing inter-individual variability rather than pathology. Third, the healthy group was on average younger and lighter than the patient group, and although the age distributions overlap substantially and the within-participant M0–M6 contrast is unaffected by these differences, the between-group comparisons are not adjusted for them. An age- and BMI-matched comparison group would directly separate the contributions of demographic differences and pathology. Fourth, our marker-based, model-free approach captures coordination at the level of segment displacement, limiting inference in two ways. It cannot identify the joint-level mechanisms underlying a given network signature because different compensatory strategies can produce similar topologies. More fundamentally, it describes kinematic outcomes rather than physiological causes, so the splinting and motor-persistence accounts remain interpretations of movement rather than demonstrations of neuromuscular mechanisms. Concurrent electromyography could test these interpretations directly. Fifth, positive and negative partial correlations are not equally easy to estimate from finite gait-cycle data—positive ones are typically more numerous and larger, making the trading-off network empirically sparser—though our prior threshold-sweep analyses (Mangalam and Kelty-Stephen, 2026) showed that the deficits are robust to the choice of sparsity threshold. Finally, replication in independent cohorts, ideally with denser longitudinal sampling across the extended recovery period, is essential before this finding grounds clinical decisions.

A further consideration, central to a systems-level analysis of distributed coordination, is concurrent musculoskeletal pathology outside the index hip. Contralateral hip degeneration, knee osteoarthritis, lumbar spondylosis or stenosis, and sacroiliac dysfunction are common in this population—particularly in older adults—and may independently shape whole-body coordination. Because the signed framework evaluates inter-segment dependencies across the entire body rather than mechanics at the operated joint, persistent abnormalities may reflect not only the index hip but ongoing compensation elsewhere in the kinetic chain. This applies most directly to the persistence of altered trading-off coordination at HOA-M6: a segmental-differentiation deficit that survives surgery on the index hip may, in some patients, reflect the integrated consequences of multi-joint disease rather than incomplete recovery of the hip itself. Persistent altered coordination architecture after THA should, therefore, not necessarily be read as surgical failure, but may instead be the integrated biomechanical signature of multi-joint disease that surgery on a single joint is not designed to address. Studies incorporating detailed characterization of adjacent-joint and spinal pathology could help disentangle hip-specific recovery from broader multisite adaptation.

In conclusion, the signed decomposition exposes a hidden layer of coordination dynamics that magnitude-based network analysis cannot detect. Recovery from hip arthroplasty is not a single trajectory but two—and at the mid-recovery time-point examined here, the one that lags, and on the one measure deteriorates, is amplitude differentiation between segments—which we take to be the mode most closely tied to fluid, non-guarded gait, though establishing that link will require studies pairing these network measures with clinical and patient-reported outcomes. By distinguishing covarying from trading-off coordination as separable network properties, this framework yields a mechanistically grounded account of how unilateral hip pathology fragments whole-body movement and how surgery partially, but asymmetrically, restores it.

## Data Availability

Publicly available datasets were analyzed in this study. These data can be found here: https://doi.org/10.6084/m9.figshare.14420645, https://doi.org/10.6084/m9.figshare.14420756.
